# The Study on Single-Event Effects and Hardening Analysis of Frequency Divider Circuits Based on InP HBT Process

**DOI:** 10.3390/mi15040527

**Published:** 2024-04-15

**Authors:** Xiaohong Zhao, Yongbo Su, You Chen, Yihao Zhang, Jianjun Xiang, Siyi Cheng, Yurong Bai

**Affiliations:** 1 School of Aviation Engineering, Air Force Engineering University, Xi’an 710051, China; 2 Institute of Microelectronics, Chinese Academy of Sciences, Beijing 100029, China; 3 School of Nuclear Science and Technology, Xi’an Jiaotong University, Xi’an 710049, China

**Keywords:** single-event effect, divider circuit, InP heterojunction bipolar transistor, harden circuit

## Abstract

The single-event effects (SEEs) of frequency divider circuits and the radiation tolerance of the hardened circuit are studied in this paper. Based on the experimental results of SEEs in InP HBTs, a transient current model for sensitive transistors is established, taking into account the influence of factors such as laser energy, base-collector junction voltage, and radiation position. Moreover, the SEEs of the (2:1) static frequency divider circuit with the InP DHBT process are simulated under different laser energies by adding the transient current model at sensitive nodes. The effect of the time relationship between the pulsed laser and clock signal are discussed. Changes in differential output voltage and the degradation mechanism of unhardened circuits are analyzed, which are mainly attributed to the cross-coupling effect between the transistors in the differential pair. Furthermore, the inverted output is directly connected to the input, leading to a feedback loop and causing significant logic upsets. Finally, an effective hardened method is proposed to provide redundancy and mitigate the impacts of SEEs on the divider. The simulation results demonstrate a notable improvement in the radiation tolerance of the divider.

## 1. Introduction

With the rapid development of wireless radio applications, circuit systems are encountering the challenge of processing vast amounts of data. This leads to an unprecedented growing demand for high-speed and high-frequency circuits, such as millimeter-wave wireless communication [1], the next-generation optical Ethernet [2], and high digital oscilloscopes [3]. Among these applications, the frequency divider circuit plays a crucial role in analog circuits, as it represents the circuit unit with the highest operating frequency. Frequency dividers are essential for converting high-frequency signals into low-frequency signals, commonly used in frequency synthesizers, orthogonal signal generation, and clock recovery circuits [4,5,6,7]. Moreover, an InP/InGaAs heterojunction bipolar transistor (HBT) excels by virtue of its high gain, mobility, breakdown voltage, and superior linearity characteristics when compared to other millimeter-wave devices, making it the preferred choice for the implementation of high-speed divider circuits. For instance, in order to achieve the same frequency, the feature size of an InP HBT is approximately two times larger than that of a SiGe HBT, along with having a higher breakdown voltage. In other words, InP HBTs can achieve much higher frequencies for the same feature size. Therefore, InP HBTs demonstrate the significant potential for applications in high-frequency circuits.

When used in a space environment, InP-based circuits are exposed to a large number of radiation particles, leading to various steady and transient radiation effects within the devices. While extensive research has been published on the total dose effects and displacement effects of InP HBT and circuits [8,9,10,11,12], this paper focuses specifically on the investigation of single-event effects (SEEs). Currently, most of the research concentrates on the SEEs and hardened methods for SiGe HBTs and Si-based CMOS circuits [13,14,15,16,17,18,19], with limited research on SEEs of InP-based circuits. For instance, T. R. Weatherford et al. simulated the SEEs of InP HBTs in emitter-coupled logic circuits and analyzed the influence of the lifetime of buffer layer materials on SEEs [20]. D. L. Hansen et al. discussed the relationship between cross-section induced by SEEs and the clock frequency in limiting amplifier circuits [21], and in another paper, they studied the SEEs and cross-section caused by protons and heavy ions in InP- and SiGe-based shift register circuits [22]. P. Chu et al. studied the impact of the heavy ion-induced SEEs on clock speed and driving voltage in InP-based shift register circuits [23]. Y. T. Zhang et al. discussed the SEEs of InP-based unhardened and hardened trigger circuits [24]. These references analyzed the effect of SEEs on InP- and SiGe-based limiting amplifier circuits and shift register circuits; however, the mechanism of SEEs in InP-based frequency divider circuits remains unclear. Thus, it is essential to delve into the single-event effects in InP-based divider circuits to analyze the radiation tolerance of circuits.

Due to the limited availability of experimental time and the unpredictable positioning of heavy ion beams within the circuit, it is difficult to precisely locate sensitive transistors through heavy ion experiments. Moreover, the analysis of SEE mechanisms mainly relies on the reverse deduction from test results. However, in this paper, a transient current model is established based on experimental results of the SEEs in InP HBTs. Furthermore, the SEEs of the unhardened and hardened broadband static divider circuit with the InP DHBT process are simulated under different laser energies by adding the transient current model at sensitive nodes. The detailed changes in the differential output voltage and degradation mechanism of unhardened circuits are analyzed. The hardened method is proposed, and the radiation tolerance of the hardened divider is discussed. The transient current model of SEEs in InP HBTs is presented in Section 2. The analysis of sensitive structures and SEEs in divider circuits is shown in Section 3. The analysis of the hardened divider circuit is in Section 4. The conclusion is given in Section 5.

## 2. Transient Current Model of SEEs in InP HBT

In the previous study, the pulsed laser-induced SEEs of InP HBTs were studied with different laser energies, the base-collector junction (BC junction) bias, and irradiation positions [25]. When the pulsed laser ir radiates InP HBTs, numerous electron-hole pairs are generated in the device. Under the influence of the electric field and the gradient of carrier concentration, the electron-hole pairs are separated and collected by electrodes with the function of drift and diffusion. It is obvious that the waveforms of the measured collector transient currents are similar to a Gaussian distribution, which is a typical model to describe the transient current induced by SEEs [26]. Therefore, the transient current model is proposed in Equation (1), which includes various influencing factors:

(1) $$I(t,E,V,x)=T_{0}+P(E,V,x)\exp\left[-2\cdot {\left(\frac{t-t_{0}}{w}\right)}^{2}\right]$$

where *T*_0_ is the transient current distribution position parameter, *t*_0_ is the center position of the current distribution, *w* is the width parameter of the Gaussian distribution, *P* is the peak value of the current distribution, *E* is the pulsed laser energy, *V* is the BC junction bias, and *x* is the distance of irradiation positions away from the center of the device.

Based on measured results of SEEs in InP HBTs [25], when the laser energy varies from 10 pJ to 100 pJ with V_CB_ = 1 V (the bias of base-collector junction) and V_BE_ = 0 V (the bias of emitter-base junction), and the irradiated position is located at 1.5 μm away from the device center, the extracted parameter values of transient current model under different laser energies are shown in Table 1. It is found that except for the peak values of the transient current (*P*), the rest of the relevant parameters, including the center moment of the transient current distribution *t*_0_, the width of the current distribution *w*, and the transient current distribution position parameter *T*_0_, remain largely consistent under different laser energies. The same phenomenon also occurs under different biases of BC junction and irradiation positions. Therefore, only the relationships between the peak value parameters (*P*) and various laser energies (*E*), the BC junction bias (*V*), and the irradiation positions (*x*) are considered.

**Figure 1:**
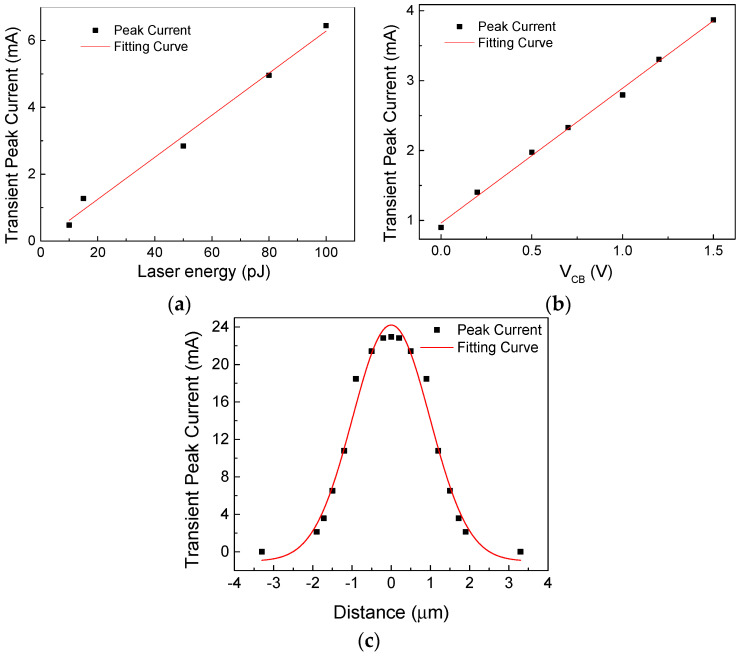
The relationship between the peak values of transient current and different influence factors: (**a**) The laser energy; (**b**) The biases of BC junction; (**c**) The irradiation position.

Figure 1a–c show the measured results and fitting curves of the transient current peak values (*P*) with different conditions. It is found that the peak values rise linearly with the increase in laser energy (*E*) and the bias of BC junction (*V*) in Figure 1a,b. From Figure 1c, when the pulsed laser irradiates at different positions, the closer to the center of the device, the greater the peak values. Thus, the nonlinear relationship between peak values and irradiation positions is fitted, which is in good agreement with the Gaussian distribution. Therefore, the expression for the peak values (*P*) is proposed in Equation (2):

(2) $$P\left(E,V,x\right)=aE+bV+c\exp\left[-2\cdot {\left(\frac{x}{197}\right)}^{2}\right]+d$$

where *a* and *b* represent the slopes of the curves in Figure 1a and Figure 1b, respectively, and *c* and *d* can be calculated by the values in Figure 1c when the laser energy and BC junction bias are constant. Consequently, the values of parameters are *a* = 6.29 × 10^−5^, *b* = 1.93 × 10^−3^, *c* = 0.025, *d* = −9.67 × 10^−3^.

**Figure 2:**
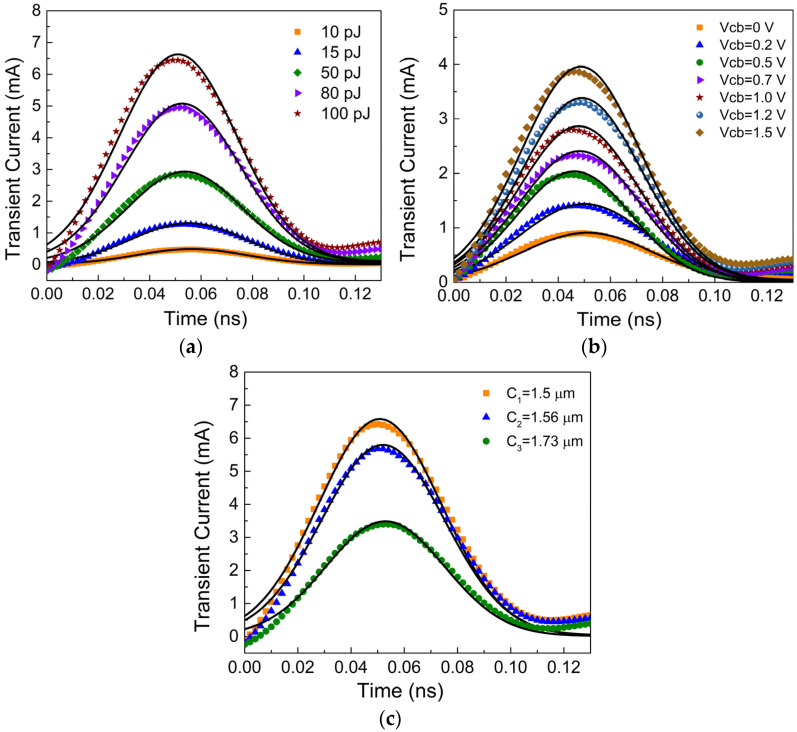
The measured and model results of collector transient current with different conditions: (**a**) The laser energy; (**b**) The collector bias; (**c**) The irradiation position.

Substituting Equation (2) into Equation (1), the transient current model *I*(*t*, *E*, *V*, *x*) of the InP HBT can be obtained. The comparison plots between the transient current model and measured results of the InP HBT are shown in Figure 2a–c. It can be seen that the transient current model can accurately characterize the impact of SEEs on the collector current of InP HBTs under different conditions. Therefore, a comprehensive analysis of SEEs in InP-based circuits can be carried out based on this model.

The relationship between the laser energy and linear energy transfer (LET) for heavy ions is shown in Table 2. Based on the Geant4 simulation software 10.4, the LET values corresponding to different laser energies are simulated and calculated for two incident particles, ^59^Ni and ^80^Br. This calculation aligns with the amount of charge collected by the collector under various laser energies and considers the emitter, base, and collector regions as sensitive areas. As can be seen in Table 2, it is evident that the LET values remain consistent for different particles.

## 3. Sensitive Structures and SEEs in Divider Circuits

### 3.1. Basic Functions of Circuits

**Figure 3:**
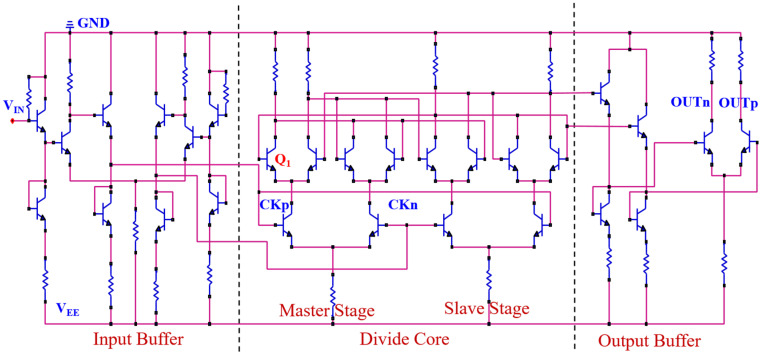
Diagram of the frequency divider circuit (The red lines represent the connecting lines; Blue lines represent electronic components).

Figure 3 shows the diagram of the 2:1 broadband static frequency divider circuit with the InP DHBT process [27,28,29]. The transient response of the circuit is simulated using RF simulation software 2019 (Advanced Design System, ADS) based on the 0.8 μm InP DHBT process provided by the Institute of Microelectronics, Chinese Academy of Sciences. The core structure consists of two latches (master and slave structure), which are connected in a series. The inverted output is then connected back to the input. The input buffer module provides differential clock signals, while the output buffer module employs an emitter follower to enhance the driving capability of output. The circuit is triggered on the rising edge of the clock, and it operates within a frequency range of 4 GHz to 58 GHz.

### 3.2. Sensitive Structures in Divider Circuits

In both the input and output buffer modules, the performance of the transistors is stable whether the base is connected to the collector, or the collector is directly grounded. In addition, when analyzed by the transient current model, it is found that the impact of SEEs on the transistor with the collector grounded through a resistor is relatively small compared to core circuit. Therefore, our focus is primarily on the core circuit of the frequency divider, which consists of a master and slave latch structure. The master stage operates in a tracking state, while the slave stage is in a hold state until the rising edge of the next clock. There are four clocked transistors in the core structure, which are always operating in the amplification state as switches. Additionally, eight transistors are utilized for data transmission. The stability of the collector voltage of these transistors plays a crucial role in achieving the logic function of the frequency divider. Therefore, they are considered as the most sensitive components in the divider circuit.

### 3.3. SEEs in the Divider Circuit

**Figure 4:**
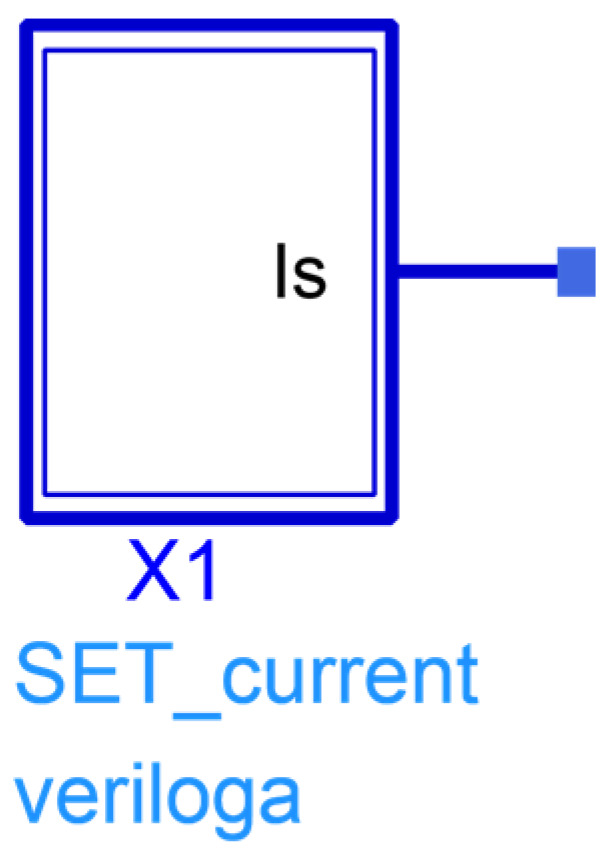
The transient current source model.

**Figure 5:**
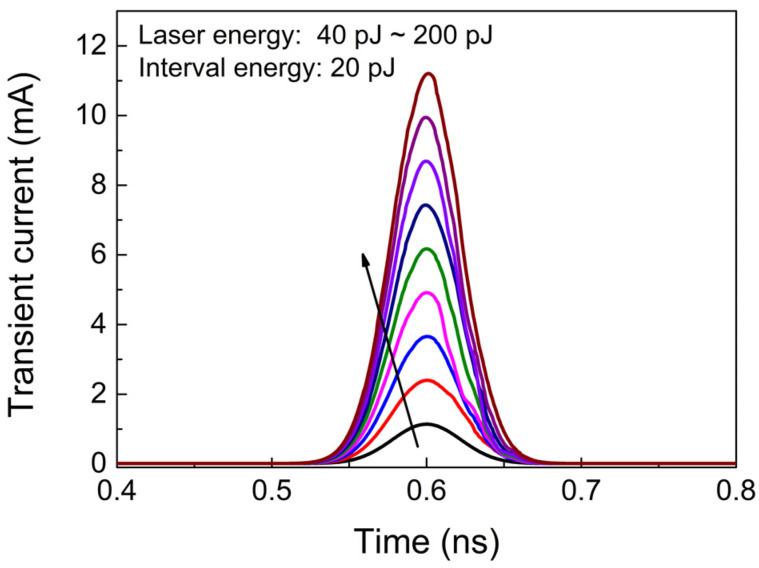
The transient current waveforms of Q_1_.

The transient current model in Equation (1) is developed by code scripting in ADS, forming a current module, as shown in Figure 4. The circuits suffering from ion strikes are simulated by adding the transient current model at sensitive nodes. Assuming that the Q_1_ transistor in the master stage is affected by a SEE, the collector transient current of Q_1_ with different laser energies is shown in Figure 5. The voltage of base-collector junction of Q_1_ is 0.21 V and the laser focal point is situated 1.5 μm away from the device center. Then, the single-event effects (SEEs) of the frequency divider circuit can be simulated by connecting the transient current module to the collector of Q1. An input signal with an amplitude of −10 dBm and a frequency of 40 GHz is used. The circuit is biased with a negative power supply and V_EE_ is set at −2.5 V.

**Figure 6:**
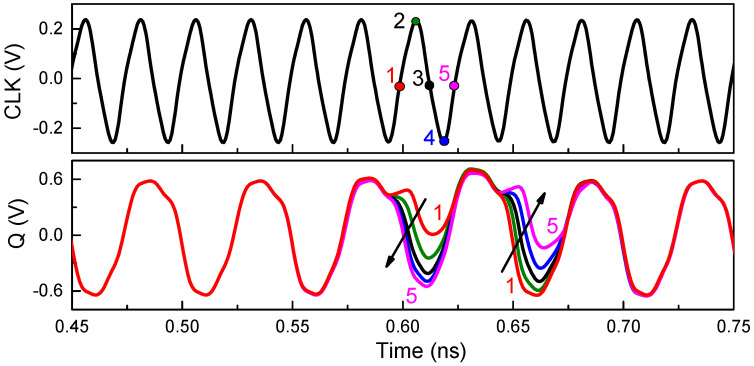
The effect of the time relationship between the pulsed laser and clock signal.

The divider reads data on the rising edge of the clock and holds these data on the falling edge. In order to study the effect of the time relationship between the pulsed laser and clock signal, the differential output Q of the divider circuit is simulated when SEEs occur at the rising edge, high level, falling edge, and low level of the clock signal, respectively, as shown in Figure 6. It is evident that the greatest effect of SEEs on the output characteristics occurs at the rising edge of the clock (position 1). Since the frequency divider is reading the data at this time, the differential voltage caused by SEEs is passed directly to the output. As the occurrence time of SEEs shifts to the right, the effect of SEEs on the output is gradually transferred to the next low level of output until the next rising edge of clock signal (position 5). Thus, it can be inferred that the SEE triggered at the rising edge of the clock is the worst case. Therefore, the center time of the transient current in Figure 5 is approximately 0.6 ns, coinciding with the rising edge of the clock signal.

**Figure 7:**
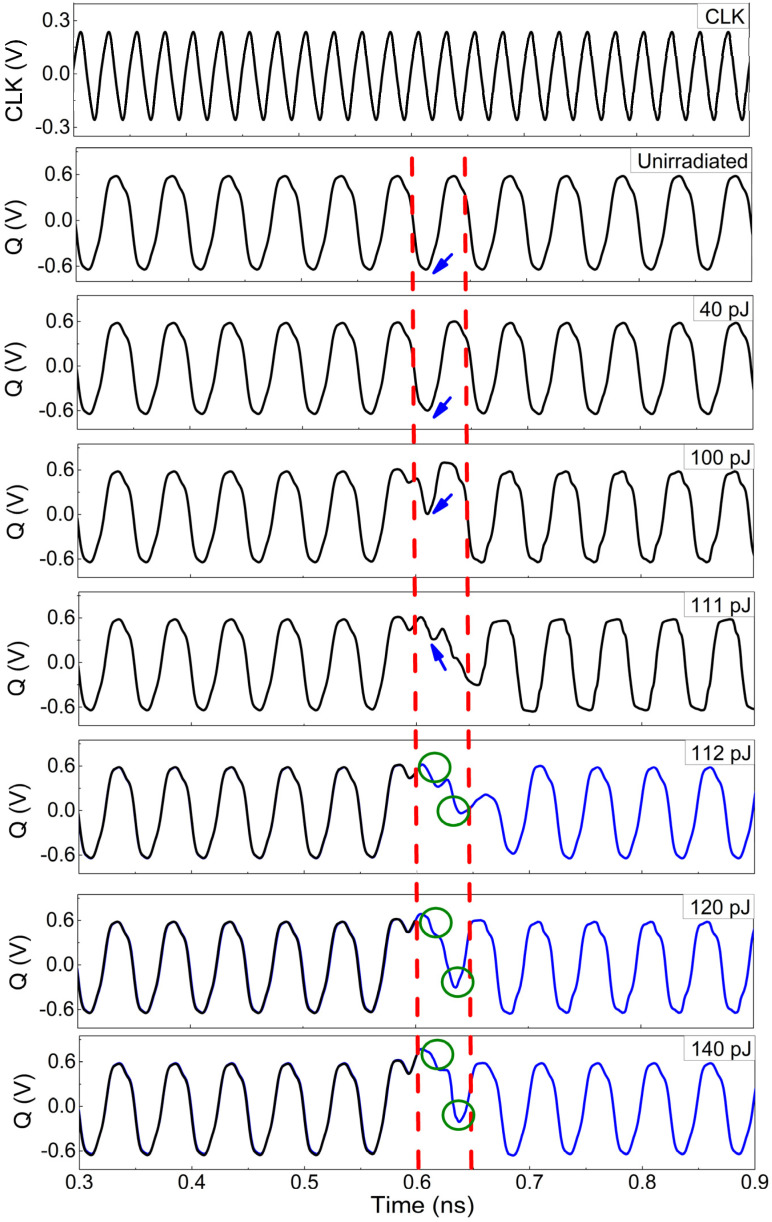
The waveforms of clock and differential output Q with un-irradiated and irradiated by different laser energies.

Figure 7 shows the differential clock and output waveforms of the un-irradiated and irradiated frequency divider. Starting from 40 pJ, the low voltage of the output gradually upsets due to SEEs (indicated by the blue arrow) until 111 pJ of laser energy. As indicated by the red dashed line in Figure 7, the impact of SEEs on the output is limited to the time interval between 0.6 ns and 0.65 ns. According to the transient current parameters in Table 1, it is known that the distribution width of the collector transient current under different conditions is about 44 ps, while the frequency of the output signal in the divider is 20 GHz, corresponding to a period of 50 ps. Therefore, the influence of the SEEs on the divider would only last for one period. However, when the laser energy exceeds 112 pJ, as depicted by the blue curves in Figure 7, the low level within the 0.6 to 0.65 ns completely flips to a high level due to the SEEs. As a result, when the next rising edge of the clock arrives, the original high level undergoes a transition to a low level (compared to the green circles), causing all outputs of the divider to flip after 0.6 ns. This transition results in significant logic errors.

**Figure 8:**
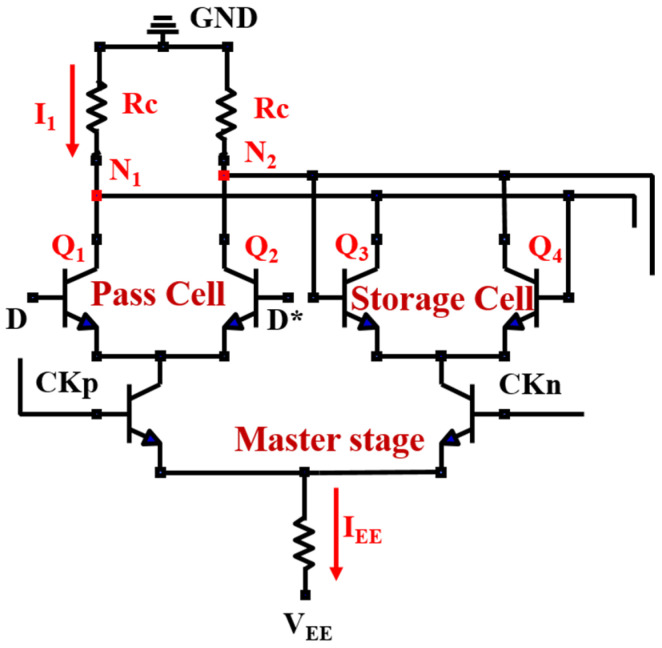
The master stage of the divider circuit (D and D* are a pair of differential inputs in the pass cell).

**Figure 9:**
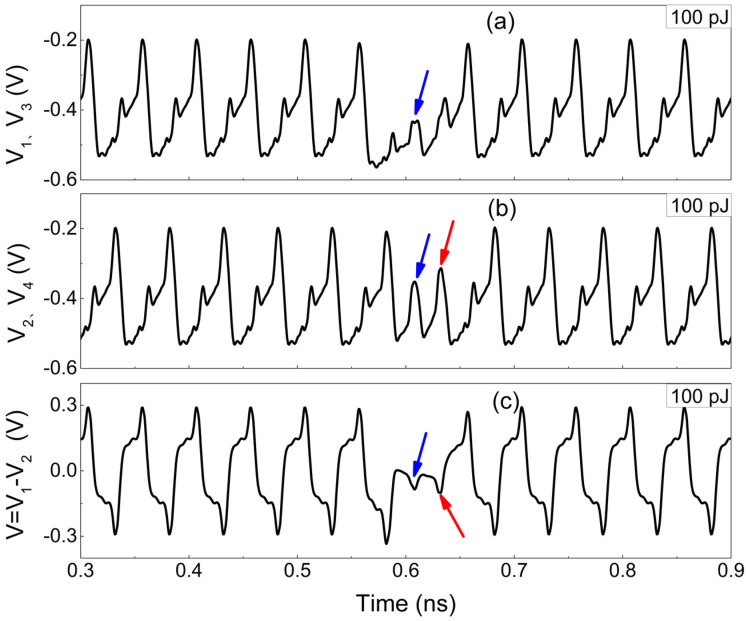
The waveforms of node voltage and differential voltage (**a**) The collector voltages of Transistor Q_1_ and Q_3_; (**b**) The collector voltages of Transistor Q_4_ and Q_3_; (**c**) The collector voltage difference between Q_1_ and Q_2_ transistors.

The changes depicted in Figure 7 are related to the operation mechanism of the frequency divider [30]. The master stage structure of the divider is shown in Figure 8, including two pairs of differential transistors for passing and storing the signal, respectively. The direction of the static tail current I_EE_ is determined by the input voltage of base in the differential transistors, which always flows towards the transistor with the higher base voltage. Thus, the collector voltage of the transistors in the differential pair is determined by the voltage drop across the resistance (Rc). The greater the current (I_1_) flowing through Rc, the lower the collector voltage (−RcI_1_) due to the negative power supply. If transistor Q_1_ suffers from a SEE, the total current in the branch where Q_1_ is located would increase due to the transient current induced by SEE. As a result, the transient voltage V_1_ at node N_1_ decreases (it was originally at a high voltage level), as shown in Figure 9a.

As seen in Figure 8, there is a cross-coupling between Q_1_ and Q_2_ (so as to Q_3_ and Q_4_). Specifically, the collector (C) of Q_1_ is connected to the base (B) of Q_4_, while the collector (C) of Q_2_ is linked to the collector (C) of Q_4_. Therefore, the decrease in the base voltage of Q_4_ (induced by V_1_) reduces current flowing through Q_4_, causing the collector voltage of Q_4_ to rise, as well as V_2_ (it was originally at a low voltage level), as indicated by the blue arrow in Figure 9b. At the arrival of the next clock’s rising edge, V_2_ is expected to flip a high voltage level. However, the increased output (in Figure 7) connected to the base of Q_2_, causes the decrease in V_2_, as indicated by the red arrow in Figure 9b. As a result, the differential voltage (V = V_1_ − V_2_) exhibits a significant decrease in the peak of the positive voltage and an increase in the peak of the negative voltage after 0.6 ns due to the opposite variations in V_1_ and V_2_, as seen in Figure 9c. 

As can be seen from Figure 3, the change in differential voltage (V) in the master stage can be transmitted to the output through the slave stage and then fed back to the input via the inverted output, creating a feedback loop. When the pulsed laser energy is less than 112 pJ, the differential voltage does not flip in Figure 9c. Thus, the logic function of outputs would not be affected by SEEs, and only the amplitude of the output voltage changes in Figure 7. As the laser energy exceeds 112 pJ, the peak of the differential voltage (indicated by the blue arrow) continues to decrease in Figure 9c, and the negative peak continues to increase (indicated by the red arrow), resulting in an inversion of the high and low levels of the differential voltage. Subsequently, as a result of the feedback loop, significant logic errors are introduced into the output signal. 

However, the cross-coupling circuit and feedback loop are indispensable for achieving the function of the divider. Therefore, it is considered to reduce the cross-coupling between differential pair transistors in pass and storage cells in order to enhance the radiation tolerance of the divider circuit.

## 4. Hardening Analysis of the Divider Circuit 

**Figure 10:**
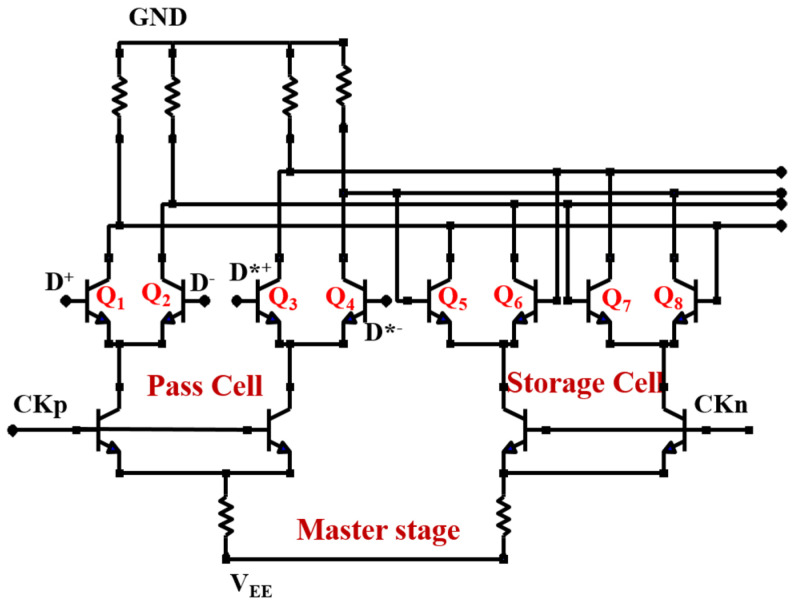
The master stage of the hardened divider circuit (D*^+^ and D*^−^ are a pair of differential inputs in the pass cell).

The hardened master stage of the divider is illustrated in Figure 10. Although the hardened circuit appears to be a simple parallel connection of the two structures in Figure 8, a careful examination reveals its ingenuity [19]. Effective decoupling is achieved between the base and collector of the differential pair transistors in pass and storage cells. For instance, the base electrodes of Q_5_ and Q_6_ in the storage cell, which were previously connected to the collectors of Q_2_ and Q_1_ in the pass cell in the unhardened circuit, are now connected to the collectors of Q_3_ and Q_4_ in the pass cell, and the collectors of Q_5_ and Q_6_ are connected to the collectors of Q_1_ and Q_2_. A similar connection method is used for Q_7_ and Q_8_.

**Figure 11:**
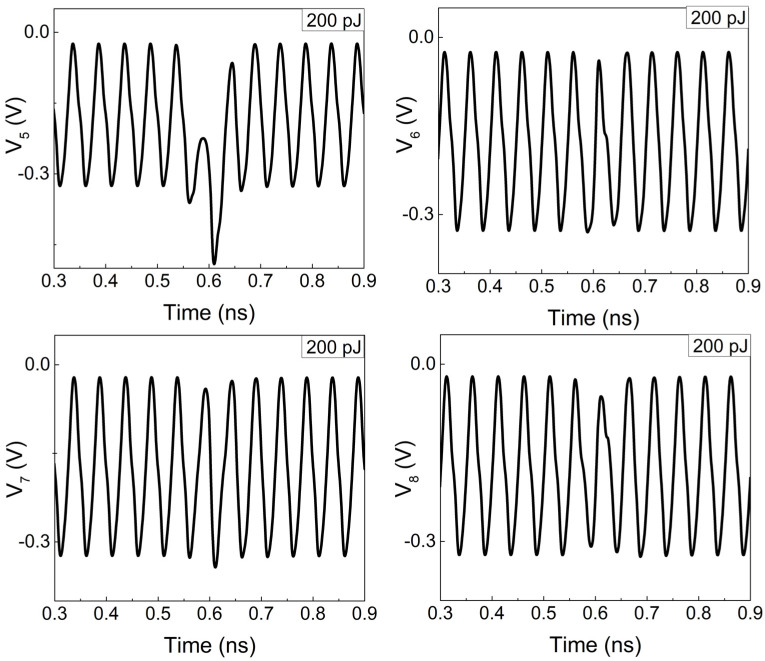
The voltage waveforms of transistor Q_5_~Q_8_.

In such circumstances, when Q_1_ experiences SEEs, only the collector of Q_5_, which is directly connected to Q_1_, is significantly affected. Conversely, the collectors of Q_6_, Q_7_, and Q_8_ indirectly connected to Q_1_ through the base are less affected. Figure 11 shows the transient waveforms of the collector voltages of Q_5_ to Q_8_ under 200 pJ pulsed laser irradiation. It is evident that the voltages of other ports remain stable except for Q_5_. Therefore, Q_5_ and Q_8_ are chosen as one differential pair, while Q_6_ and Q_7_ are considered as another pair to transmit the signal to the slave stage and outputs. This configuration provides a degree of redundancy against SEEs, which ensures that at least one of the differential outputs is relatively less affected. 

**Figure 12:**
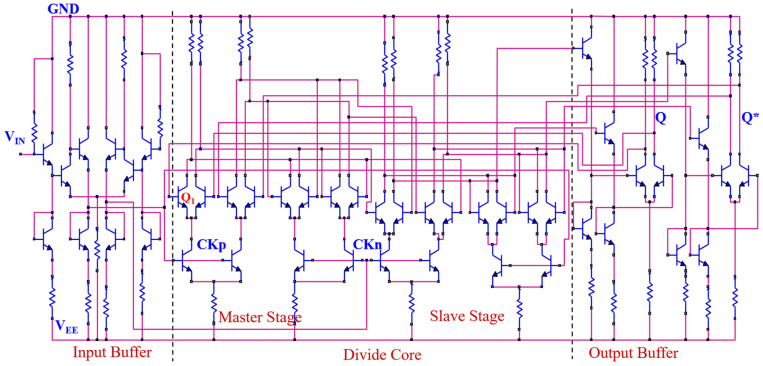
The hardened divider circuit (The red lines represent the connecting lines; Blue lines represent electronic components).

A similar hardened method is also applied to the slave stage and output buffer structure. The diagram of the hardened divider is shown in Figure 12. Furthermore, the performance of the unhardened and hardened divider is summarized and compared with some previously reported dividers in Table 3. It is found that the hardened static frequency divider proposed in this paper demonstrates competitively lower power dissipation.

**Figure 13:**
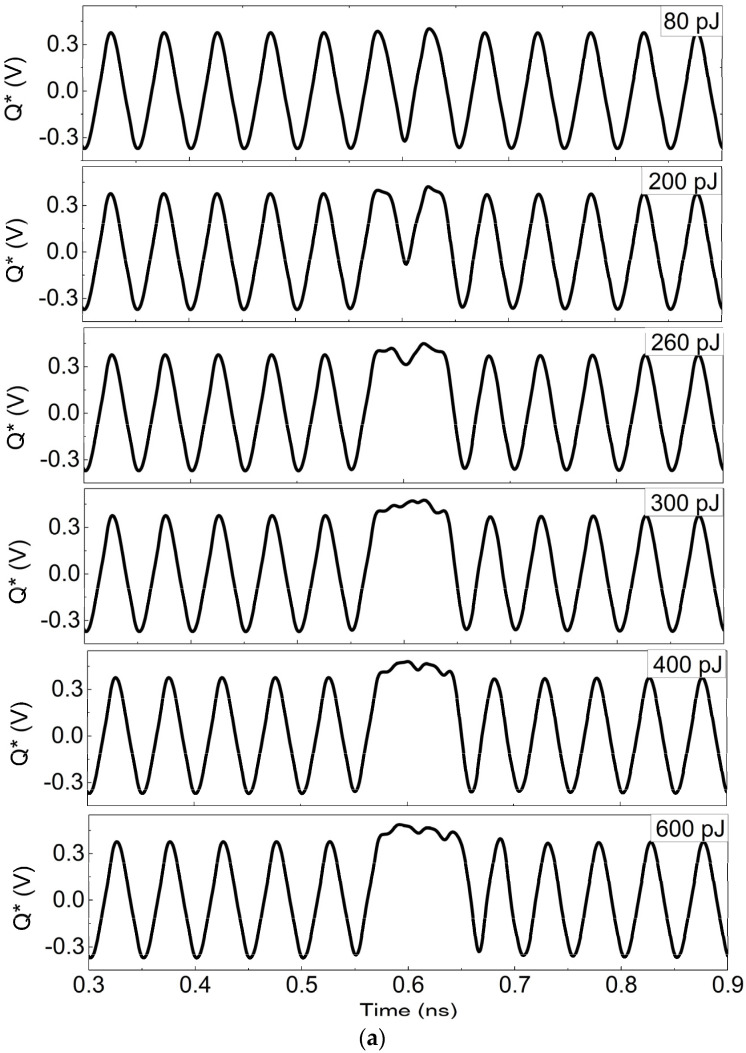

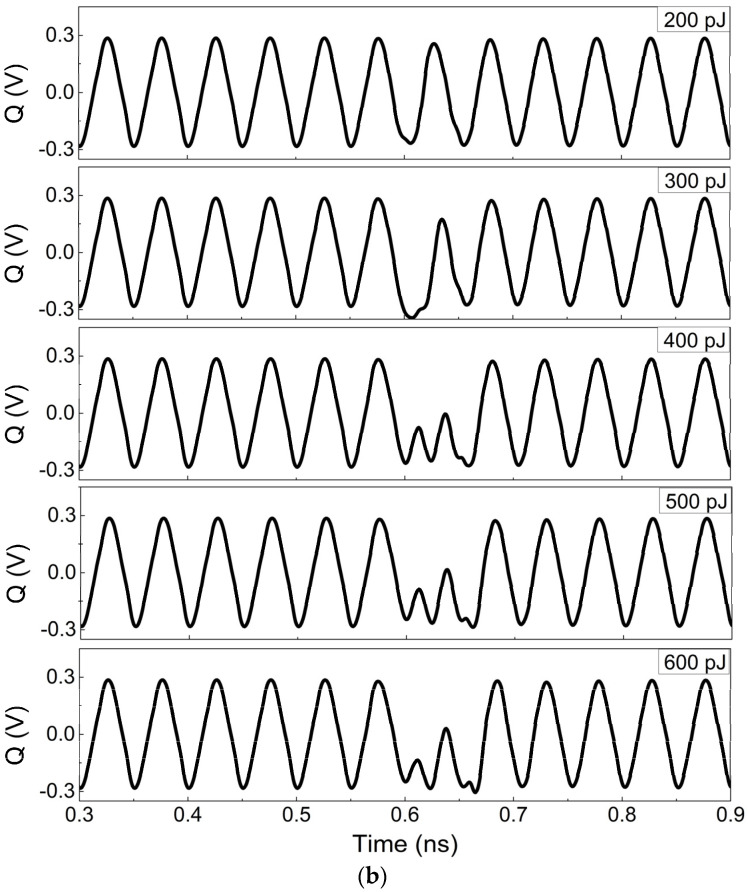
Differential output Q* and Q waveforms of the hardened circuit irradiated by different laser energies (**a**) Q*; (**b**) Q.

The simulation results of differential outputs Q* and Q with different laser energies in the hardened frequency divider are shown in Figure 13a,b. Compared to Figure 7, on the one hand, the differential output Q* of hardened circuit in Figure 13a exhibits a gradual change from 80 pJ of laser energy, and the low voltage flips after 260 pJ. In this circumstance, only a single bit flips even when laser energy is beyond 600 pJ, without triggering the flipping of all data. On the other hand, what is more important in Figure 13b is that no flipping occurs in another differential output Q until 600 pJ and the logic function is unaffected by SEEs. Only the voltage amplitude gradually decreases starting from 200 pJ. It is mainly attributed to the effective decoupling of the differential pair transistors. In addition, the differential outputs in the hardened dividers play a crucial role in offering complementary adjustments to the input, effectively preventing significant logic errors. Therefore, it is indicated that at least one of the differential outputs in the hardened divider can maintain the correct logic function, which significantly improves the circuit’s radiation tolerance to SEEs and provides reliable performance.

## 5. Conclusions

In this paper, the SEEs of the unhardened and hardened broadband static divider circuit with the InP DHBT process are studied under different laser energies by adding the transient current model at sensitive nodes. The core structure of the frequency divider consisting of master and slave stages is considered to be a sensitive structure. The effect of the time relationship between the pulsed laser and clock signal is discussed. It can be inferred that the SEE triggered at the rising edge of the clock is the worst case. The significant variation in the differential voltage caused by the SEE is mainly attributed to the cross-coupling effect between the differential pair transistors. When the laser energy is less than 112 pJ, the differential voltage remains stable, and the impact of SEEs on the amplitude of the divider output is minimal. Thus, the logic function of outputs would not be affected by SEEs. However, when the laser energy exceeds 112 pJ, an upset of the high and low levels of the differential voltage is induced, causing significant logic upsets in the subsequent outputs due to the feedback loop. Based on the analysis of SEE mechanisms in the frequency divider circuit, an effective hardened method is proposed to decouple the differential pair transistors. Two basic units are connected in parallel to generate dual differential outputs, providing redundancy for SEEs. Simulation results show that one of the differential outputs is less affected by SEEs, ensuring the normal logic function of the divider and greatly improving the radiation tolerance of the circuit to SEEs.

## Figures and Tables

**Table 1:** The transient current model parameter values with different laser energies.

Laser Energy (E)	*T*_0_	*t*_0_	*w*	*P*
10 pJ	9.62 × 10^−6^ A	6.10 × 10^−11^ s	4.31 × 10^−11^ s	4.82 × 10^−4^ A
15 pJ	9.61 × 10^−6^ A	6.05 × 10^−11^ s	4.46 × 10^−11^ s	0.00129 A
50 pJ	9.62 × 10^−6^ A	6.04 × 10^−11^ s	4.43 × 10^−11^ s	0.00287 A
80 pJ	9.63 × 10^−6^ A	6.03 × 10^−11^ s	4.51 × 10^−11^ s	0.00499 A
100 pJ	9.63 × 10^−6^ A	6.10 × 10^−11^ s	4.57 × 10^−11^ s	0.00653 A

**Table 2:** The LET corresponding to different laser energies.

		Incident Ions			
		^59^Ni		^80^Br	
Laser Energy/pJ	Collected Charge/fC	Energy/MeV	LET/MeV/cm^2^/mg	Energy/MeV	LET/MeV/cm^2^/mg
10	31.89	1.1	3.63	1.1	3.83
15	82.61	2.5	7.29	2.5	7.53
50	222.49	8	18.47	8	19.07
80	331.62	15	28.52	15	28.84
100	416.31	24	36.33	21	36.59

**Table 3:** Comparison with some previously reported dividers.

References	Process	The Highest Input Frequency/GHz	The Number of Transistors	Power Dissipation/mW
Ref. [30]	0.7 μm InP DHBTs	83	30	620
Ref. [31]	0.7 μm InP DHBTs	48	42	264
Ref. [32]	0.7 μm InP DHBTs	43	42	230
Ref. [33]	0.8 μm InP DHBTs	62	22	380
Ref. [34]	0.8 μm InP DHBTs	40	30	650
Ref. [28]	0.8 μm InP DHBTs	62	34	382
Ref. [35]	0.8 μm InP DHBTs	66	42	424
The unhardened circuit in this paper	0.8 μm InP DHBTs	58	28	219
The hardened circuit in this paper	0.8 μm InP DHBTs	50	44	348

## Data Availability

The original contributions presented in the study are included in the article, further inquiries can be directed to the corresponding author.
